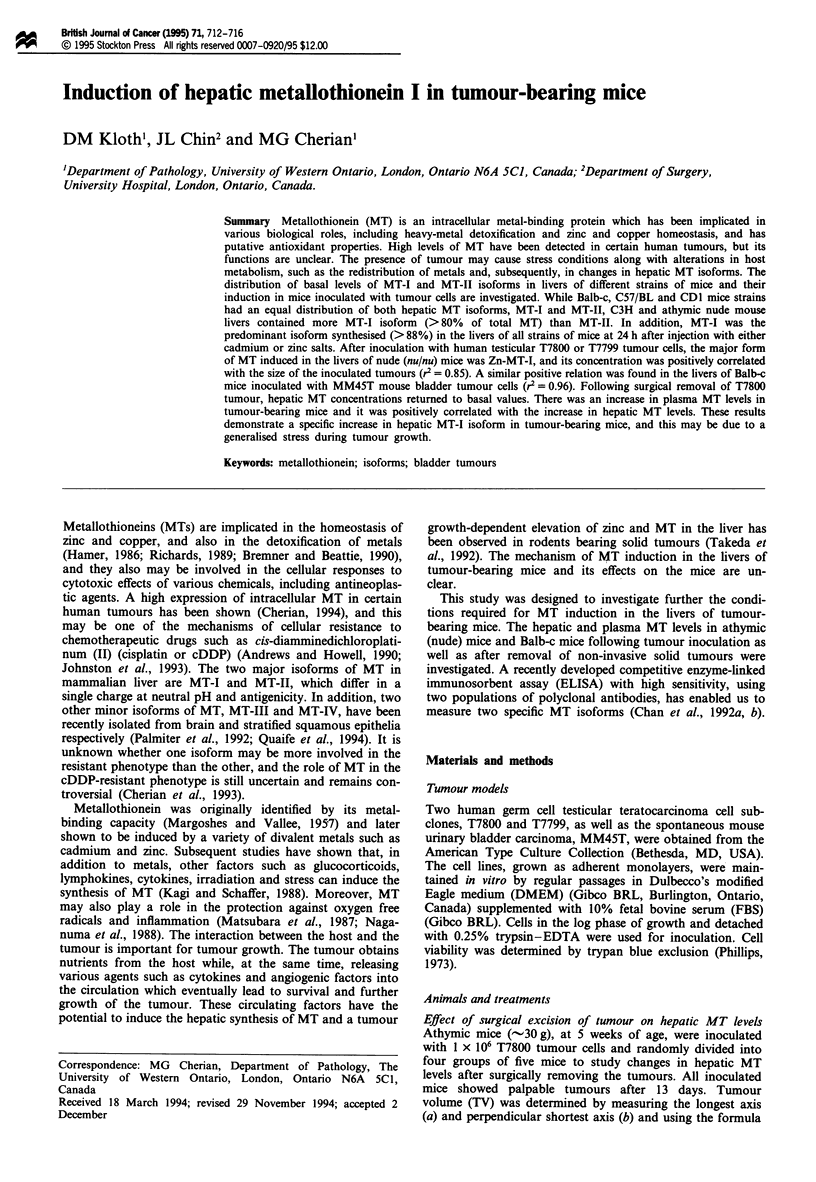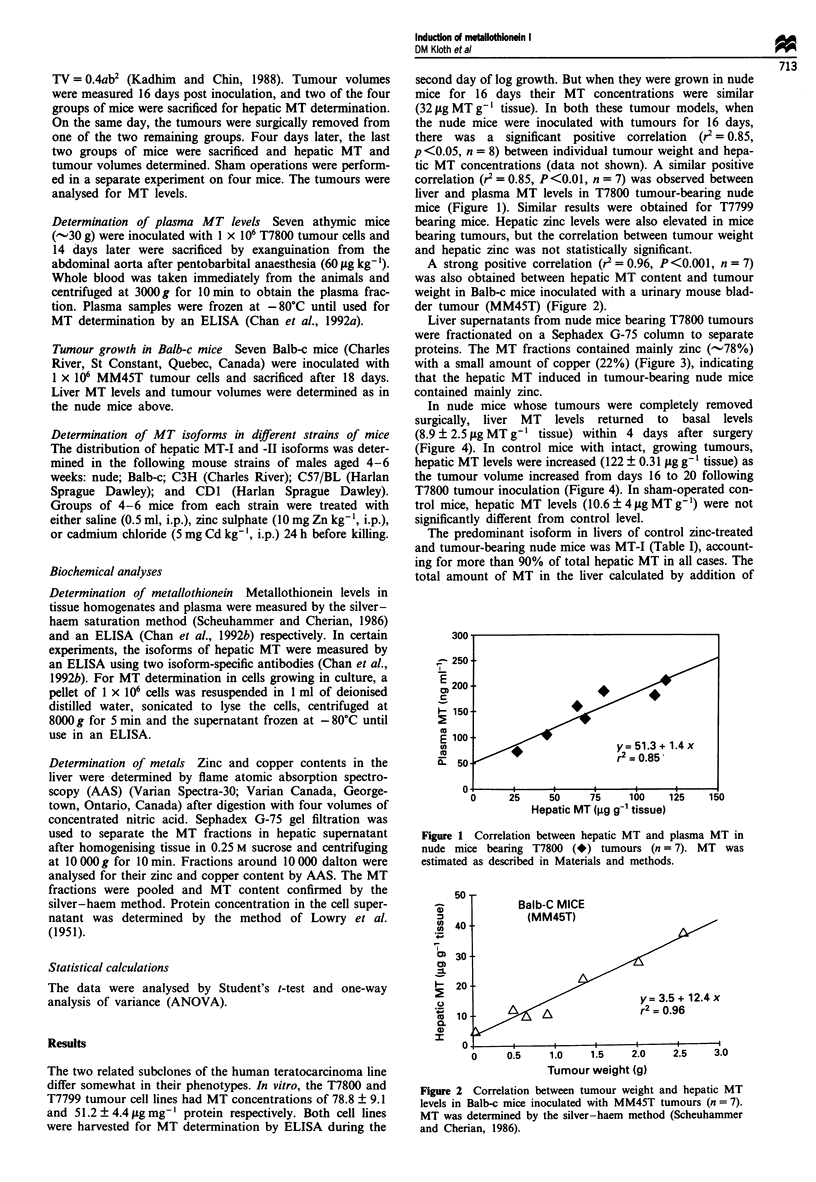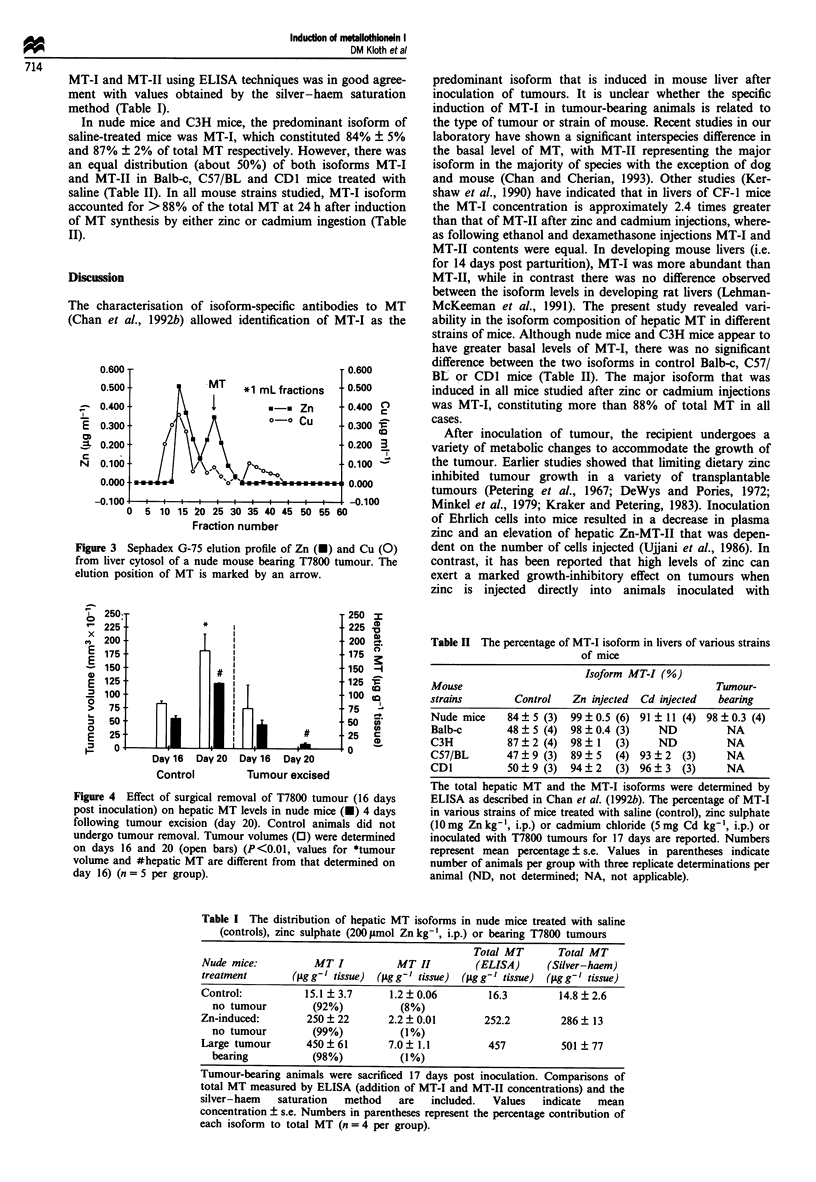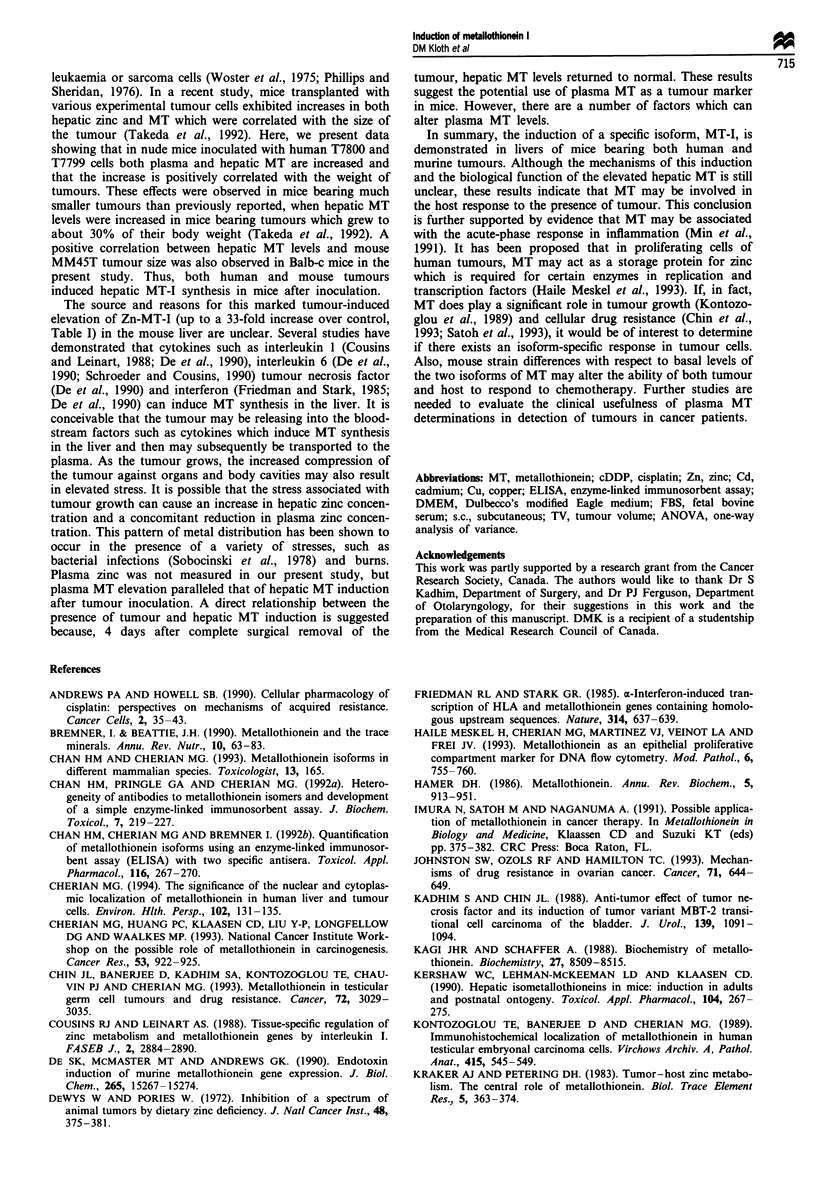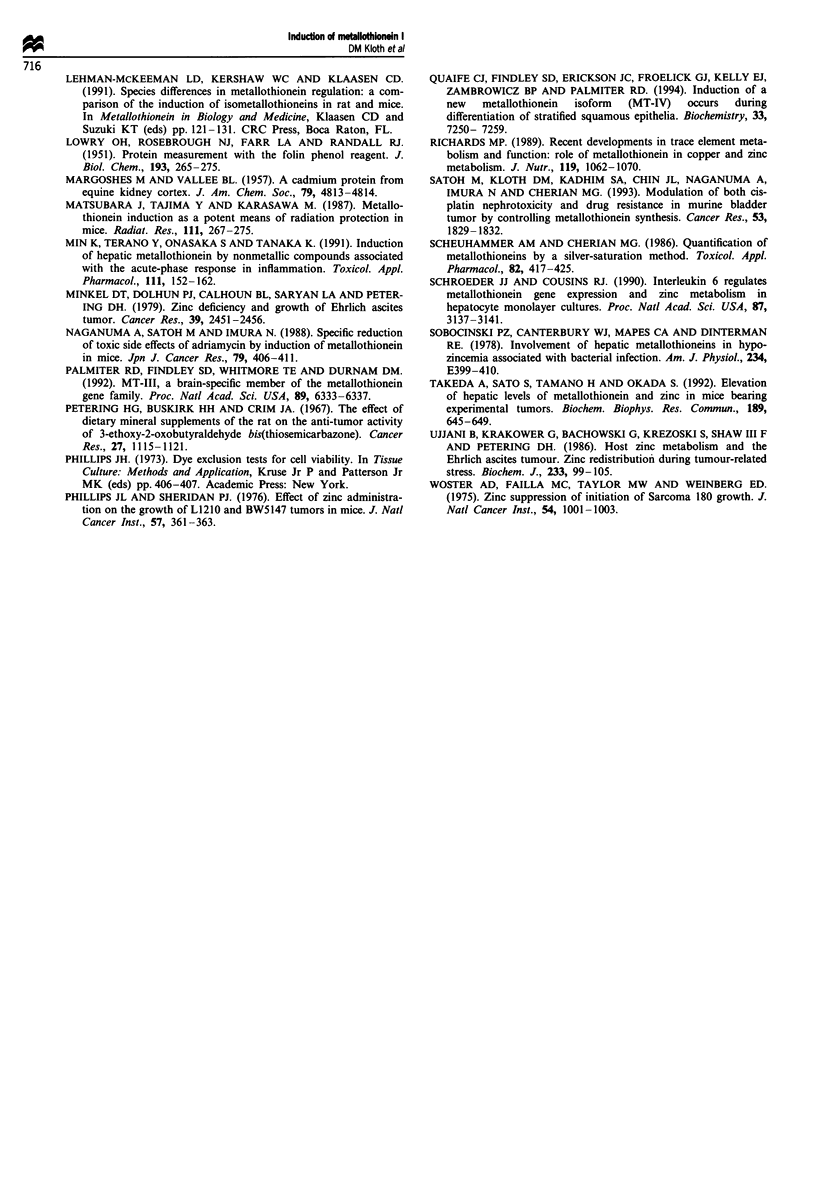# Induction of hepatic metallothionein I in tumour-bearing mice.

**DOI:** 10.1038/bjc.1995.139

**Published:** 1995-04

**Authors:** D. M. Kloth, J. L. Chin, M. G. Cherian

**Affiliations:** Department of Pathology, University of Western Ontario, London, Canada.

## Abstract

Metallothionein (MT) is an intracellular metal-binding protein which has been implicated in various biological roles, including heavy-metal detoxification and zinc and copper homeostasis, and has putative antioxidant properties. High levels of MT have been detected in certain human tumours, but its functions are unclear. The presence of tumour may cause stress conditions along with alterations in host metabolism, such as the redistribution of metals and, subsequently, in changes in hepatic MT isoforms. The distribution of basal levels of MT-1 and MT-11 isoforms in livers of different strains of mice and their induction in mice inoculated with tumour cells are investigated. While Balb-c, C57/BL and CD1 mice strains had an equal distribution of both hepatic MT isoforms, MT-I and MT-II. In addition, MT-I was the predominant isoform synthesised (> 88%) in the livers of all strains of mice at 24 h after injection with either cadmium or zinc salts. After inoculation with human testicular T7800 or T7799 tumour cells, the major form of MT induced in the livers of nude (nu/nu) mice was Zn-MT-I, and its concentration was positively correlated with the size of the inoculated tumours (r2 = 0.85). A similar positive relation was found in the livers of Balb-c mice inoculated with MM45T mouse bladder tumour cells (r2 = 0.96). Following surgical removal of T7800 tumour, hepatic MT concentrations returned to basal values. There was an increase in plasma MT levels in tumour-bearing mice and it was positively correlated with the increase in hepatic MT levels. These results demonstrate a specific increase in hepatic MT-I isoform in tumour-bearing mice, and this may be due to a generalised stress during tumour growth.


British Journal of Cancer (1995) 71, 712-716
© 1995 Stockton Press All rights reserved 0007-0920/95 $12.00

### Induction of hepatic metallothionein I in tumour-bearing mice

DM Kloth[1], JL Chin[2] and MG Cherian[1]

[1]*Department of Pathology, University of Western Ontario, London, Ontario N6A 5C1, Canada;* [2]*Department of Surgery, University Hospital, London, Ontario, Canada.*

**Summary** Metallothionein (MT) is an intracellular metal-binding protein which has been implicated in various biological roles, including heavy-metal detoxification and zinc and copper homeostasis, and has putative antioxidant properties. High levels of MT have been detected in certain human tumours, but its functions are unclear. The presence of tumour may cause stress conditions along with alterations in host metabolism, such as the redistribution of metals and, subsequently, in changes in hepatic MT isoforms. The distribution of basal levels of MT-I and MT-II isoforms in livers of different strains of mice and their induction in mice inoculated with tumour cells are investigated. While Balb-c, C57/BL and CD1 mice strains had an equal distribution of both hepatic MT isoforms, MT-I and MT-II, C3H and athymic nude mouse livers contained more MT-I isoform (>80% of total MT) than MT-II. In addition, MT-I was the predominant isoform synthesised (>88%) in the livers of all strains of mice at 24 h after injection with either cadmium or zinc salts. After inoculation with human testicular T7800 or T7799 tumour cells, the major form of MT induced in the livers of nude (*nu/nu*) mice was Zn-MT-I, and its concentration was positively correlated with the size of the inoculated tumours ($r^2 = 0.85$). A similar positive relation was found in the livers of Balb-c mice inoculated with MM45T mouse bladder tumour cells ($r^2 = 0.96$). Following surgical removal of T7800 tumour, hepatic MT concentrations returned to basal values. There was an increase in plasma MT levels in tumour-bearing mice and it was positively correlated with the increase in hepatic MT levels. These results demonstrate a specific increase in hepatic MT-I isoform in tumour-bearing mice, and this may be due to a generalised stress during tumour growth.

**Keywords:** metallothionein; isoforms; bladder tumours

Metallothioneins (MTs) are implicated in the homeostasis of zinc and copper, and also in the detoxification of metals (Hamer, 1986; Richards, 1989; Bremner and Beattie, 1990), and they also may be involved in the cellular responses to cytotoxic effects of various chemicals, including antineoplastic agents. A high expression of intracellular MT in certain human tumours has been shown (Cherian, 1994), and this may be one of the mechanisms of cellular resistance to chemotherapeutic drugs such as *cis*-diamminedichloroplatinum (II) (cisplatin or cDDP) (Andrews and Howell, 1990; Johnston *et al.*, 1993). The two major isoforms of MT in mammalian liver are MT-I and MT-II, which differ in a single charge at neutral pH and antigenicity. In addition, two other minor isoforms of MT, MT-III and MT-IV, have been recently isolated from brain and stratified squamous epithelia respectively (Palmiter *et al.*, 1992; Quaife *et al.*, 1994). It is unknown whether one isoform may be more involved in the resistant phenotype than the other, and the role of MT in the cDDP-resistant phenotype is still uncertain and remains controversial (Cherian *et al.*, 1993).

Metallothionein was originally identified by its metal-binding capacity (Margoshes and Vallee, 1957) and later shown to be induced by a variety of divalent metals such as cadmium and zinc. Subsequent studies have shown that, in addition to metals, other factors such as glucocorticoids, lymphokines, cytokines, irradiation and stress can induce the synthesis of MT (Kagi and Schaffer, 1988). Moreover, MT may also play a role in the protection against oxygen free radicals and inflammation (Matsubara *et al.*, 1987; Naganuma *et al.*, 1988). The interaction between the host and the tumour is important for tumour growth. The tumour obtains nutrients from the host while, at the same time, releasing various agents such as cytokines and angiogenic factors into the circulation which eventually lead to survival and further growth of the tumour. These circulating factors have the potential to induce the hepatic synthesis of MT and a tumour growth-dependent elevation of zinc and MT in the liver has been observed in rodents bearing solid tumours (Takeda *et al.*, 1992). The mechanism of MT induction in the livers of tumour-bearing mice and its effects on the mice are unclear.

This study was designed to investigate further the conditions required for MT induction in the livers of tumour-bearing mice. The hepatic and plasma MT levels in athymic (nude) mice and Balb-c mice following tumour inoculation as well as after removal of non-invasive solid tumours were investigated. A recently developed competitive enzyme-linked immunosorbent assay (ELISA) with high sensitivity, using two populations of polyclonal antibodies, has enabled us to measure two specific MT isoforms (Chan *et al.*, 1992*a*, *b*).

Correspondence: MG Cherian, Department of Pathology, The University of Western Ontario, London, Ontario N6A 5C1, Canada
Received 18 March 1994; revised 29 November 1994; accepted 2 December

### Materials and methods

### *Tumour models*

Two human germ cell testicular teratocarcinoma cell subclones, T7800 and T7799, as well as the spontaneous mouse urinary bladder carcinoma, MM45T, were obtained from the American Type Culture Collection (Bethesda, MD, USA). The cell lines, grown as adherent monolayers, were maintained *in vitro* by regular passages in Dulbecco's modified Eagle medium (DMEM) (Gibco BRL, Burlington, Ontario, Canada) supplemented with 10% fetal bovine serum (FBS) (Gibco BRL). Cells in the log phase of growth and detached with 0.25% trypsin-EDTA were used for inoculation. Cell viability was determined by trypan blue exclusion (Phillips, 1973).

### *Animals and treatments*

*Effect of surgical excision of tumour on hepatic MT levels* Athymic mice (~30 g), at 5 weeks of age, were inoculated with $1 \times 10^6$ T7800 tumour cells and randomly divided into four groups of five mice to study changes in hepatic MT levels after surgically removing the tumours. All inoculated mice showed palpable tumours after 13 days. Tumour volume (TV) was determined by measuring the longest axis (*a*) and perpendicular shortest axis (*b*) and using the formula

TV = $0.4ab^2$ (Kadhim and Chin, 1988). Tumour volumes were measured 16 days post inoculation, and two of the four groups of mice were sacrificed for hepatic MT determination. On the same day, the tumours were surgically removed from one of the two remaining groups. Four days later, the last two groups of mice were sacrificed and hepatic MT and tumour volumes determined. Sham operations were performed in a separate experiment on four mice. The tumours were analysed for MT levels.

*Determination of plasma MT levels* Seven athymic mice (~30 g) were inoculated with $1 \times 10^6$ T7800 tumour cells and 14 days later were sacrificed by exanguination from the abdominal aorta after pentobarbital anaesthesia (60 $\mu g\ kg^{-1}$). Whole blood was taken immediately from the animals and centrifuged at 3000 *g* for 10 min to obtain the plasma fraction. Plasma samples were frozen at −80°C until used for MT determination by an ELISA (Chan *et al.*, 1992*a*).

*Tumour growth in Balb-c mice* Seven Balb-c mice (Charles River, St Constant, Quebec, Canada) were inoculated with $1 \times 10^6$ MM45T tumour cells and sacrificed after 18 days. Liver MT levels and tumour volumes were determined as in the nude mice above.

*Determination of MT isoforms in different strains of mice* The distribution of hepatic MT-I and -II isoforms was determined in the following mouse strains of males aged 4–6 weeks: nude; Balb-c; C3H (Charles River); C57/BL (Harlan Sprague Dawley); and CD1 (Harlan Sprague Dawley). Groups of 4–6 mice from each strain were treated with either saline (0.5 ml, i.p.), zinc sulphate (10 mg Zn $kg^{-1}$, i.p.), or cadmium chloride (5 mg Cd $kg^{-1}$, i.p.) 24 h before killing.

### Biochemical analyses

*Determination of metallothionein* Metallothionein levels in tissue homogenates and plasma were measured by the silver-haem saturation method (Scheuhammer and Cherian, 1986) and an ELISA (Chan *et al.*, 1992*b*) respectively. In certain experiments, the isoforms of hepatic MT were measured by an ELISA using two isoform-specific antibodies (Chan *et al.*, 1992*b*). For MT determination in cells growing in culture, a pellet of $1 \times 10^6$ cells was resuspended in 1 ml of deionised distilled water, sonicated to lyse the cells, centrifuged at 8000 *g* for 5 min and the supernatant frozen at −80°C until use in an ELISA.

*Determination of metals* Zinc and copper contents in the liver were determined by flame atomic absorption spectroscopy (AAS) (Varian Spectra-30; Varian Canada, Georgetown, Ontario, Canada) after digestion with four volumes of concentrated nitric acid. Sephadex G-75 gel filtration was used to separate the MT fractions in hepatic supernatant after homogenising tissue in 0.25 M sucrose and centrifuging at 10 000 *g* for 10 min. Fractions around 10 000 dalton were analysed for their zinc and copper content by AAS. The MT fractions were pooled and MT content confirmed by the silver-haem method. Protein concentration in the cell supernatant was determined by the method of Lowry *et al.* (1951).

### Statistical calculations

The data were analysed by Student's *t*-test and one-way analysis of variance (ANOVA).

### Results

The two related subclones of the human teratocarcinoma line differ somewhat in their phenotypes. *In vitro*, the T7800 and T7799 tumour cell lines had MT concentrations of 78.8 ± 9.1 and 51.2 ± 4.4 $\mu g\ mg^{-1}$ protein respectively. Both cell lines were harvested for MT determination by ELISA during the second day of log growth. But when they were grown in nude mice for 16 days their MT concentrations were similar (32 $\mu g$ MT $g^{-1}$ tissue). In both these tumour models, when the nude mice were inoculated with tumours for 16 days, there was a significant positive correlation ($r^2 = 0.85$, $p < 0.05$, $n = 8$) between individual tumour weight and hepatic MT concentrations (data not shown). A similar positive correlation ($r^2 = 0.85$, $P < 0.01$, $n = 7$) was observed between liver and plasma MT levels in T7800 tumour-bearing nude mice (Figure 1). Similar results were obtained for T7799 bearing mice. Hepatic zinc levels were also elevated in mice bearing tumours, but the correlation between tumour weight and hepatic zinc was not statistically significant.

A strong positive correlation ($r^2 = 0.96$, $P < 0.001$, $n = 7$) was also obtained between hepatic MT content and tumour weight in Balb-c mice inoculated with a urinary mouse bladder tumour (MM45T) (Figure 2).

Liver supernatants from nude mice bearing T7800 tumours were fractionated on a Sephadex G-75 column to separate proteins. The MT fractions contained mainly zinc (~78%) with a small amount of copper (22%) (Figure 3), indicating that the hepatic MT induced in tumour-bearing nude mice contained mainly zinc.

In nude mice whose tumours were completely removed surgically, liver MT levels returned to basal levels (8.9 ± 2.5 $\mu g$ MT $g^{-1}$ tissue) within 4 days after surgery (Figure 4). In control mice with intact, growing tumours, hepatic MT levels were increased (122 ± 0.31 $\mu g\ g^{-1}$ tissue) as the tumour volume increased from days 16 to 20 following T7800 tumour inoculation (Figure 4). In sham-operated control mice, hepatic MT levels (10.6 ± 4 $\mu g$ MT $g^{-1}$) were not significantly different from control level.

The predominant isoform in livers of control zinc-treated and tumour-bearing nude mice was MT-I (Table I), accounting for more than 90% of total hepatic MT in all cases. The total amount of MT in the liver calculated by addition of

**Figure 1** Correlation between hepatic MT and plasma MT in nude mice bearing T7800 (◆) tumours ($n = 7$). MT was estimated as described in Materials and methods.

**Figure 2** Correlation between tumour weight and hepatic MT levels in Balb-c mice inoculated with MM45T tumours ($n = 7$). MT was determined by the silver-haem method (Scheuhammer and Cherian, 1986).

MT-I and MT-II using ELISA techniques was in good agreement with values obtained by the silver–haem saturation method (Table I).

In nude mice and C3H mice, the predominant isoform of saline-treated mice was MT-I, which constituted 84% ± 5% and 87% ± 2% of total MT respectively. However, there was an equal distribution (about 50%) of both isoforms MT-I and MT-II in Balb-c, C57/BL and CD1 mice treated with saline (Table II). In all mouse strains studied, MT-I isoform accounted for >88% of the total MT at 24 h after induction of MT synthesis by either zinc or cadmium ingestion (Table II).

### Discussion

The characterisation of isoform-specific antibodies to MT (Chan *et al.*, 1992*b*) allowed identification of MT-I as the predominant isoform that is induced in mouse liver after inoculation of tumours. It is unclear whether the specific induction of MT-I in tumour-bearing animals is related to the type of tumour or strain of mouse. Recent studies in our laboratory have shown a significant interspecies difference in the basal level of MT, with MT-II representing the major isoform in the majority of species with the exception of dog and mouse (Chan and Cherian, 1993). Other studies (Kershaw *et al.*, 1990) have indicated that in livers of CF-1 mice the MT-I concentration is approximately 2.4 times greater than that of MT-II after zinc and cadmium injections, whereas following ethanol and dexamethasone injections MT-I and MT-II contents were equal. In developing mouse livers (i.e. for 14 days post parturition), MT-I was more abundant than MT-II, while in contrast there was no difference observed between the isoform levels in developing rat livers (Lehman-McKeeman *et al.*, 1991). The present study revealed variability in the isoform composition of hepatic MT in different strains of mice. Although nude mice and C3H mice appear to have greater basal levels of MT-I, there was no significant difference between the two isoforms in control Balb-c, C57/BL or CD1 mice (Table II). The major isoform that was induced in all mice studied after zinc or cadmium injections was MT-I, constituting more than 88% of total MT in all cases.

After inoculation of tumour, the recipient undergoes a variety of metabolic changes to accommodate the growth of the tumour. Earlier studies showed that limiting dietary zinc inhibited tumour growth in a variety of transplantable tumours (Petering *et al.*, 1967; DeWys and Pories, 1972; Minkel *et al.*, 1979; Kraker and Petering, 1983). Inoculation of Ehrlich cells into mice resulted in a decrease in plasma zinc and an elevation of hepatic Zn-MT-II that was dependent on the number of cells injected (Ujjani *et al.*, 1986). In contrast, it has been reported that high levels of zinc can exert a marked growth-inhibitory effect on tumours when zinc is injected directly into animals inoculated with

**Figure 3** Sephadex G-75 elution profile of Zn (■) and Cu (○) from liver cytosol of a nude mouse bearing T7800 tumour. The elution position of MT is marked by an arrow.

**Figure 4** Effect of surgical removal of T7800 tumour (16 days post inoculation) on hepatic MT levels in nude mice (■) 4 days following tumour excision (day 20). Control animals did not undergo tumour removal. Tumour volumes (□) were determined on days 16 and 20 (open bars) ($P<0.01$, values for *tumour volume and #hepatic MT are different from that determined on day 16) ($n = 5$ per group).

**Table II** The percentage of MT-I isoform in livers of various strains of mice

| *Mouse strains* | *Control* | *Zn injected* | *Cd injected* | *Tumour-bearing* |
|---|---|---|---|---|
| | *Isoform MT-I (%)* | | | |
| Nude mice | 84 ± 5 (3) | 99 ± 0.5 (6) | 91 ± 11 (4) | 98 ± 0.3 (4) |
| Balb-c | 48 ± 5 (4) | 98 ± 0.4 (3) | ND | NA |
| C3H | 87 ± 2 (4) | 98 ± 1 (3) | ND | NA |
| C57/BL | 47 ± 9 (3) | 89 ± 5 (4) | 93 ± 2 (3) | NA |
| CD1 | 50 ± 9 (3) | 94 ± 2 (3) | 96 ± 3 (3) | NA |

The total hepatic MT and the MT-I isoforms were determined by ELISA as described in Chan *et al.* (1992*b*). The percentage of MT-I in various strains of mice treated with saline (control), zinc sulphate (10 mg Zn kg$^{-1}$, i.p.) or cadmium chloride (5 mg Cd kg$^{-1}$, i.p.) or inoculated with T7800 tumours for 17 days are reported. Numbers represent mean percentage ± s.e. Values in parentheses indicate number of animals per group with three replicate determinations per animal (ND, not determined; NA, not applicable).

**Table I** The distribution of hepatic MT isoforms in nude mice treated with saline (controls), zinc sulphate (200 µmol Zn kg$^{-1}$, i.p.) or bearing T7800 tumours

| *Nude mice: treatment* | *MT I (µg g$^{-1}$ tissue)* | *MT II (µg g$^{-1}$ tissue)* | *Total MT (ELISA) (µg g$^{-1}$ tissue)* | *Total MT (Silver–haem) (µg g$^{-1}$ tissue)* |
|---|---|---|---|---|
| Control: no tumour | 15.1 ± 3.7 (92%) | 1.2 ± 0.06 (8%) | 16.3 | 14.8 ± 2.6 |
| Zn-induced: no tumour | 250 ± 22 (99%) | 2.2 ± 0.01 (1%) | 252.2 | 286 ± 13 |
| Large tumour bearing | 450 ± 61 (98%) | 7.0 ± 1.1 (1%) | 457 | 501 ± 77 |

Tumour-bearing animals were sacrificed 17 days post inoculation. Comparisons of total MT measured by ELISA (addition of MT-I and MT-II concentrations) and the silver–haem saturation method are included. Values indicate mean concentration ± s.e. Numbers in parentheses represent the percentage contribution of each isoform to total MT ($n = 4$ per group).

leukaemia or sarcoma cells (Woster et al., 1975; Phillips and Sheridan, 1976). In a recent study, mice transplanted with various experimental tumour cells exhibited increases in both hepatic zinc and MT which were correlated with the size of the tumour (Takeda et al., 1992). Here, we present data showing that in nude mice inoculated with human T7800 and T7799 cells both plasma and hepatic MT are increased and that the increase is positively correlated with the weight of tumours. These effects were observed in mice bearing much smaller tumours than previously reported, when hepatic MT levels were increased in mice bearing tumours which grew to about 30% of their body weight (Takeda et al., 1992). A positive correlation between hepatic MT levels and mouse MM45T tumour size was also observed in Balb-c mice in the present study. Thus, both human and mouse tumours induced hepatic MT-I synthesis in mice after inoculation.

The source and reasons for this marked tumour-induced elevation of Zn-MT-I (up to a 33-fold increase over control, Table I) in the mouse liver are unclear. Several studies have demonstrated that cytokines such as interleukin 1 (Cousins and Leinart, 1988; De et al., 1990), interleukin 6 (De et al., 1990; Schroeder and Cousins, 1990) tumour necrosis factor (De et al., 1990) and interferon (Friedman and Stark, 1985; De et al., 1990) can induce MT synthesis in the liver. It is conceivable that the tumour may be releasing into the bloodstream factors such as cytokines which induce MT synthesis in the liver and then may subsequently be transported to the plasma. As the tumour grows, the increased compression of the tumour against organs and body cavities may also result in elevated stress. It is possible that the stress associated with tumour growth can cause an increase in hepatic zinc concentration and a concomitant reduction in plasma zinc concentration. This pattern of metal distribution has been shown to occur in the presence of a variety of stresses, such as bacterial infections (Sobocinski et al., 1978) and burns. Plasma zinc was not measured in our present study, but plasma MT elevation paralleled that of hepatic MT induction after tumour inoculation. A direct relationship between the presence of tumour and hepatic MT induction is suggested because, 4 days after complete surgical removal of the tumour, hepatic MT levels returned to normal. These results suggest the potential use of plasma MT as a tumour marker in mice. However, there are a number of factors which can alter plasma MT levels.

In summary, the induction of a specific isoform, MT-I, is demonstrated in livers of mice bearing both human and murine tumours. Although the mechanisms of this induction and the biological function of the elevated hepatic MT is still unclear, these results indicate that MT may be involved in the host response to the presence of tumour. This conclusion is further supported by evidence that MT may be associated with the acute-phase response in inflammation (Min et al., 1991). It has been proposed that in proliferating cells of human tumours, MT may act as a storage protein for zinc which is required for certain enzymes in replication and transcription factors (Haile Meskel et al., 1993). If, in fact, MT does play a significant role in tumour growth (Kontozoglou et al., 1989) and cellular drug resistance (Chin et al., 1993; Satoh et al., 1993), it would be of interest to determine if there exists an isoform-specific response in tumour cells. Also, mouse strain differences with respect to basal levels of the two isoforms of MT may alter the ability of both tumour and host to respond to chemotherapy. Further studies are needed to evaluate the clinical usefulness of plasma MT determinations in detection of tumours in cancer patients.

**Abbreviations:** MT, metallothionein; cDDP, cisplatin; Zn, zinc; Cd, cadmium; Cu, copper; ELISA, enzyme-linked immunosorbent assay; DMEM, Dulbecco's modified Eagle medium; FBS, fetal bovine serum; s.c., subcutaneous; TV, tumour volume; ANOVA, one-way analysis of variance.

**Acknowledgements**
This work was partly supported by a research grant from the Cancer Research Society, Canada. The authors would like to thank Dr S Kadhim, Department of Surgery, and Dr PJ Ferguson, Department of Otolaryngology, for their suggestions in this work and the preparation of this manuscript. DMK is a recipient of a studentship from the Medical Research Council of Canada.

**References**

ANDREWS PA AND HOWELL SB. (1990). Cellular pharmacology of cisplatin: perspectives on mechanisms of acquired resistance. *Cancer Cells*, **2**, 35–43.

BREMNER, I. & BEATTIE, J.H. (1990). Metallothionein and the trace minerals. *Annu. Rev. Nutr.*, **10**, 63–83.

CHAN HM AND CHERIAN MG. (1993). Metallothionein isoforms in different mammalian species. *Toxicologist*, **13**, 165.

CHAN HM, PRINGLE GA AND CHERIAN MG. (1992*a*). Heterogeneity of antibodies to metallothionein isomers and development of a simple enzyme-linked immunosorbent assay. *J. Biochem. Toxicol.*, **7**, 219–227.

CHAN HM, CHERIAN MG AND BREMNER I. (1992*b*). Quantification of metallothionein isoforms using an enzyme-linked immunosorbent assay (ELISA) with two specific antisera. *Toxicol. Appl. Pharmacol.*, **116**, 267–270.

CHERIAN MG. (1994). The significance of the nuclear and cytoplasmic localization of metallothionein in human liver and tumour cells. *Environ. Hlth. Persp.*, **102**, 131–135.

CHERIAN MG, HUANG PC, KLAASEN CD, LIU Y-P, LONGFELLOW DG AND WAALKES MP. (1993). National Cancer Institute Workshop on the possible role of metallothionein in carcinogenesis. *Cancer Res.*, **53**, 922–925.

CHIN JL, BANERJEE D, KADHIM SA, KONTOZOGLOU TE, CHAUVIN PJ AND CHERIAN MG. (1993). Metallothionein in testicular germ cell tumours and drug resistance. *Cancer*, **72**, 3029–3035.

COUSINS RJ AND LEINART AS. (1988). Tissue-specific regulation of zinc metabolism and metallothionein genes by interleukin I. *FASEB J.*, **2**, 2884–2890.

DE SK, MCMASTER MT AND ANDREWS GK. (1990). Endotoxin induction of murine metallothionein gene expression. *J. Biol. Chem.*, **265**, 15267–15274.

DEWYS W AND PORIES W. (1972). Inhibition of a spectrum of animal tumors by dietary zinc deficiency. *J. Natl Cancer Inst.*, **48**, 375–381.

FRIEDMAN RL AND STARK GR. (1985). α-Interferon-induced transcription of HLA and metallothionein genes containing homologous upstream sequences. *Nature*, **314**, 637–639.

HAILE MESKEL H, CHERIAN MG, MARTINEZ VJ, VEINOT LA AND FREI JV. (1993). Metallothionein as an epithelial proliferative compartment marker for DNA flow cytometry. *Mod. Pathol.*, **6**, 755–760.

HAMER DH. (1986). Metallothionein. *Annu. Rev. Biochem.*, **5**, 913–951.

IMURA N, SATOH M AND NAGANUMA A. (1991). Possible application of metallothionein in cancer therapy. In *Metallothionein in Biology and Medicine*, Klaassen CD and Suzuki KT (eds) pp. 375–382. CRC Press: Boca Raton, FL.

JOHNSTON SW, OZOLS RF AND HAMILTON TC. (1993). Mechanisms of drug resistance in ovarian cancer. *Cancer*, **71**, 644–649.

KADHIM S AND CHIN JL. (1988). Anti-tumor effect of tumor necrosis factor and its induction of tumor variant MBT-2 transitional cell carcinoma of the bladder. *J. Urol.*, **139**, 1091–1094.

KAGI JHR AND SCHAFFER A. (1988). Biochemistry of metallothionein. *Biochemistry*, **27**, 8509–8515.

KERSHAW WC, LEHMAN-MCKEEMAN LD AND KLAASEN CD. (1990). Hepatic isometallothioneins in mice: induction in adults and postnatal ontogeny. *Toxicol. Appl. Pharmacol.*, **104**, 267–275.

KONTOZOGLOU TE, BANERJEE D AND CHERIAN MG. (1989). Immunohistochemical localization of metallothionein in human testicular embryonal carcinoma cells. *Virchows Archiv. A, Pathol. Anat.*, **415**, 545–549.

KRAKER AJ AND PETERING DH. (1983). Tumor-host zinc metabolism. The central role of metallothionein. *Biol. Trace Element Res.*, **5**, 363–374.

LEHMAN-MCKEEMAN LD, KERSHAW WC AND KLAASEN CD. (1991). Species differences in metallothionein regulation: a comparison of the induction of isometallothioneins in rat and mice. In *Metallothionein in Biology and Medicine*, Klaasen CD and Suzuki KT (eds) pp. 121–131. CRC Press, Boca Raton, FL.

LOWRY OH, ROSEBROUGH NJ, FARR LA AND RANDALL RJ. (1951). Protein measurement with the folin phenol reagent. *J. Biol. Chem.*, **193**, 265–275.

MARGOSHES M AND VALLEE BL. (1957). A cadmium protein from equine kidney cortex. *J. Am. Chem. Soc.*, **79**, 4813–4814.

MATSUBARA J, TAJIMA Y AND KARASAWA M. (1987). Metallothionein induction as a potent means of radiation protection in mice. *Radiat. Res.*, **111**, 267–275.

MIN K, TERANO Y, ONASAKA S AND TANAKA K. (1991). Induction of hepatic metallothionein by nonmetallic compounds associated with the acute-phase response in inflammation. *Toxicol. Appl. Pharmacol.*, **111**, 152–162.

MINKEL DT, DOLHUN PJ, CALHOUN BL, SARYAN LA AND PETERING DH. (1979). Zinc deficiency and growth of Ehrlich ascites tumor. *Cancer Res.*, **39**, 2451–2456.

NAGANUMA A, SATOH M AND IMURA N. (1988). Specific reduction of toxic side effects of adriamycin by induction of metallothionein in mice. *Jpn J. Cancer Res.*, **79**, 406–411.

PALMITER RD, FINDLEY SD, WHITMORE TE AND DURNAM DM. (1992). MT-III, a brain-specific member of the metallothionein gene family. *Proc. Natl Acad. Sci. USA*, **89**, 6333–6337.

PETERING HG, BUSKIRK HH AND CRIM JA. (1967). The effect of dietary mineral supplements of the rat on the anti-tumor activity of 3-ethoxy-2-oxobutyraldehyde *bis*(thiosemicarbazone). *Cancer Res.*, **27**, 1115–1121.

PHILLIPS JH. (1973). Dye exclusion tests for cell viability. In *Tissue Culture: Methods and Application*, Kruse Jr P and Patterson Jr MK (eds) pp. 406–407. Academic Press: New York.

PHILLIPS JL AND SHERIDAN PJ. (1976). Effect of zinc administration on the growth of L1210 and BW5147 tumors in mice. *J. Natl Cancer Inst.*, **57**, 361–363.

QUAIFE CJ, FINDLEY SD, ERICKSON JC, FROELICK GJ, KELLY EJ, ZAMBROWICZ BP AND PALMITER RD. (1994). Induction of a new metallothionein isoform (MT-IV) occurs during differentiation of stratified squamous epithelia. *Biochemistry*, **33**, 7250– 7259.

RICHARDS MP. (1989). Recent developments in trace element metabolism and function: role of metallothionein in copper and zinc metabolism. *J. Nutr.*, **119**, 1062–1070.

SATOH M, KLOTH DM, KADHIM SA, CHIN JL, NAGANUMA A, IMURA N AND CHERIAN MG. (1993). Modulation of both cisplatin nephrotoxicity and drug resistance in murine bladder tumor by controlling metallothionein synthesis. *Cancer Res.*, **53**, 1829–1832.

SCHEUHAMMER AM AND CHERIAN MG. (1986). Quantification of metallothioneins by a silver-saturation method. *Toxicol. Appl. Pharmacol.*, **82**, 417–425.

SCHROEDER JJ AND COUSINS RJ. (1990). Interleukin 6 regulates metallothionein gene expression and zinc metabolism in hepatocyte monolayer cultures. *Proc. Natl Acad. Sci. USA*, **87**, 3137–3141.

SOBOCINSKI PZ, CANTERBURY WJ, MAPES CA AND DINTERMAN RE. (1978). Involvement of hepatic metallothioneins in hypozincemia associated with bacterial infection. *Am. J. Physiol.*, **234**, E399–410.

TAKEDA A, SATO S, TAMANO H AND OKADA S. (1992). Elevation of hepatic levels of metallothionein and zinc in mice bearing experimental tumors. *Biochem. Biophys. Res. Commun.*, **189**, 645–649.

UJJANI B, KRAKOWER G, BACHOWSKI G, KREZOSKI S, SHAW III F AND PETERING DH. (1986). Host zinc metabolism and the Ehrlich ascites tumour. Zinc redistribution during tumour-related stress. *Biochem. J.*, **233**, 99–105.

WOSTER AD, FAILLA MC, TAYLOR MW AND WEINBERG ED. (1975). Zinc suppression of initiation of Sarcoma 180 growth. *J. Natl Cancer Inst.*, **54**, 1001–1003.